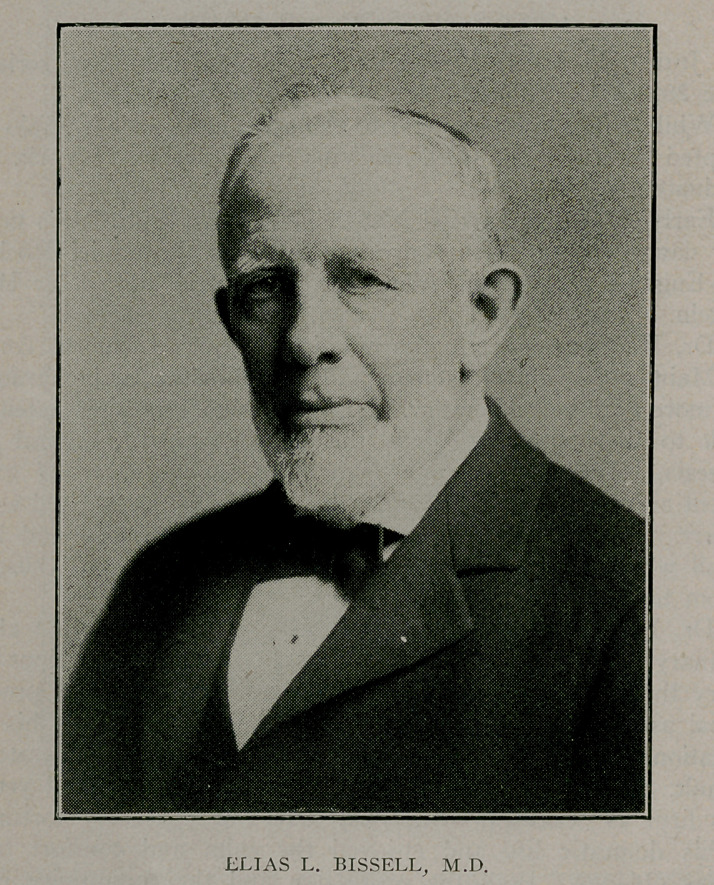# Medical Society of the County of Erie

**Published:** 1905-12

**Authors:** Francis M. O’Gorman


### SOCIETY PROCEEDINGS.

### Medical Society of the County of Erie.

*Memorial Meeting, November 29, 1905.*

REPORTED BY FRANCIS M. O'GORMAN, M. D., Secretary Pro. Tem.

Dr. A. H. Briggs, vice-president, called the Society to order at 4 P. M., and stated the object of the meeting, which was to pay tribute to three recently deceased members who had occupied distinguished places in the profession and the community.

Remarks were made by Drs. Van Peyma, Wetmore, Howe, Potter, Rochester, Snow, Smith, Briggs, Brownell, and O'Gorman.

The following memorials were adopted:

### IN MEMORIAM, DEVILLO W. HARRINGTON, M. D.,

BY LUCIAN HOWE, M. D., Buffalo.

In the death of Dr. Devillo W. Harrington this society has lost a valued member and the city of Buffalo a good citizen. His career furnishes a conspicuous example of the fulfillment of the higher possibilities in the medical profession. From the hills where his life arose, down to the sea of activities in the city where it was spent, that life has brought comfort and strength and refreshment to the many to whom he ministered. We feel that our love of country and professional zeal have been stimulated by his example. When a boy he dedicated himself to the service of his native land. He went from his country home to bear a man's part in the campaigns of the Civil War. At its

DEVILLO W. HARRINGTON, M.D.

close, although still a boy in years, he was a veteran soldier. Then, with only his hands to help him and with the scant education obtained in the little red school house, he began to build his own future. But in the struggle of professional life he grew rapidly strong, always working, always studying. He became one of the leaders in the medical profession of this vicinity. He was as gentle in the sickroom as he had been bold on the field of battle. A man of infinite tact, of commanding presence and of great kindness of heart, he was as successful in winning the sympathies and confidence of his patients, as he was astute in the affairs of the world.

The splendid gift which he has made to the Buffalo General Hospital is but the lengthened shadow of his generous service to humanity, and will stand as the happy memorial of a brave soldier, a large hearted citizen, and an honored physician.

---

### In Memoriam, John Hauenstein, M. D., 1821-1905.

By P. W. Van Peyma, M. D., of Buffale.

Dr. John Hauenstein was born at Regenfelden, Switzerland, June 28, 1821. He was the son of Jacob Hauenstein.

While still a boy he came to America and made Buffalo his adopted home. He studied medicine in Geneva, New York, and graduated in 1844.

Early in life he married Madeleine Sigwatt and of this union two daughters and two sons survive—Mrs. Nathaniel Rochester, Eugenia Hauenstein, Alfred G. Hauenstein and Oscar Hauenstein.

Dr. Hauenstein began the practice of medicine at the corner of Main and Mohawk streets and continued in active practice for more than half a century. His professional memory extended back to the days preceeding the employment of general anesthesia. During very many years his practice was of great extent and especially in obstetrics he came to be recognised as an expert. He possessed the sincere respect, admiration and love of all who knew him and this was particularly true of those to whom he ministered professionally.

Dr. Hauenstein's chief characteristic was honesty, not alone ordinary business integrity, but honesty in all his relations—in every thought and deed. He scorned self-assertion and professional assumption—he abhorred sham and pretense. In his association with his professional confrères he was considerate to a fault. His temperament was cheerful and friendly and yet he was firm as occassion demanded. He was especially fortunate in his domestic life and enjoyed the calm and comfort of a serene old age. Both the profession and the community have been elevated by his life among us.

---

### In Memoriam, Elias L. Bissell, M. D., 1835-1905.

By William Warren Potter, M. D., Buffalo.

Dr. Elias L. Bissell, of Buffalo, died at his home of disease of the heart, November 1, 1905, aged 72 years. He had suffered ill health for several years but at last his death seemed sudden, as he did not appear worse than usual up to the last moment of his life.

Dr. Bissell was a native of Erie county, having been born at Lancaster in 1833, where he received his early education. He graduated in medicine from the University of Michigan in 1861, and was commissioned assistant surgeon of the 44th infantry New York Volunteers, October 11, 1861. He was promoted surgeon of the 22nd New York Infantry, November 20, 1862,

ELIAS L. BISSELL, M.D.

and was mustered out with the regiment June 19, 1863, this being a two years' regiment. Upon his return he took up his residence at 2793 Main street, Buffalo, where he continued in medical practice until compelled to relinquish it by reason of ill health. He is survived by two daughters, both married and residing in Buffalo, and a brother, Anthony Bissell, of Lancaster. His wife died some years ago.

This brief announcement conveys but little information concerning the professional life of one of the oldest and most conscientious physicians in this great community. When Dr. Bissell established himself on North Main Street the location was well out in the suburbs, and his practice was largely in the country. This entailed all the hardships of rural medical practice, which means an incessant demand upon a man's physical resources as well as upon his mental equipment.

Dr. Bissell met these demands with a devotion to duty rarely equalled and never excelled. His great mental equipoise, though severely taxed, was never upset, and he closed a long professional career with the calm satisfaction of one who has performed his part well in the drama of life. To few men is it given to meet the requirements of an active professional career of half a century in duration, who have fulfilled the exigencies of such a life in a more satisfactory maner and with such amiability of temper.

## Figures and Tables

**Figure f1:**
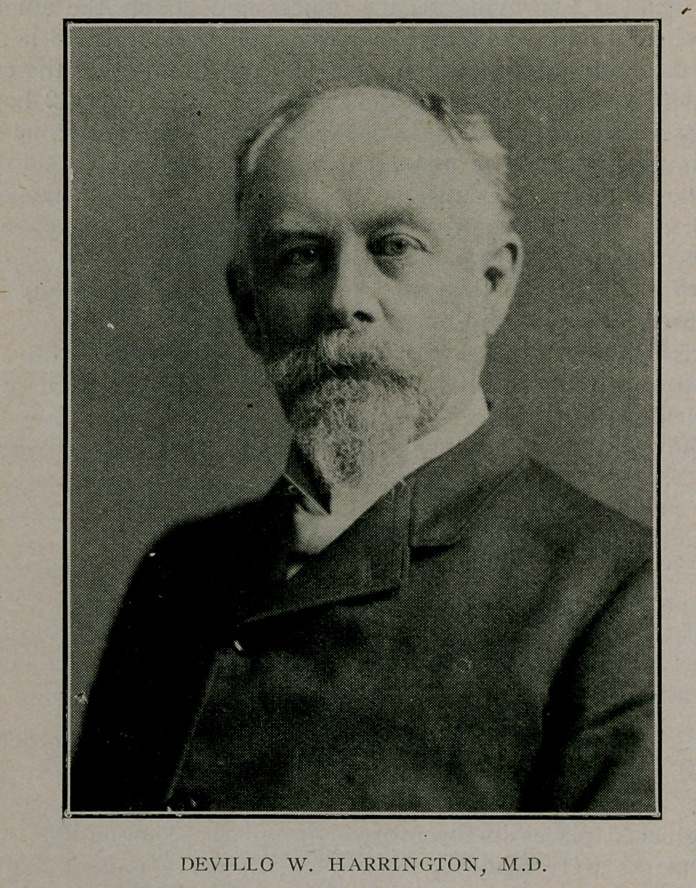


**Figure f2:**